# The Use of Maximum Entropy to Enhance Wave Intensity Analysis: An Application to Coronary Arteries in Hypertrophic Obstructive Cardiomyopathy

**DOI:** 10.3389/fcvm.2021.701267

**Published:** 2021-08-27

**Authors:** Nadine Francis, Peter P. Selwanos, Magdi H. Yacoub, Kim H. Parker

**Affiliations:** ^1^Biomedical Engineering and Innovation Laboratory, Department of Research, Aswan Heart Centre, Magdi Yacoub Heart Foundation, Aswan, Egypt; ^2^Department of Bioengineering, Imperial College, London, United Kingdom; ^3^Department of Cardiology, Aswan Heart Centre, Magdi Yacoub Heart Foundation, Aswan, Egypt; ^4^Harefield Heart Science Centre, Harefield, United Kingdom; ^5^Department of Surgery, Aswan Heart Centre, Magdi Yacoub Heart Foundation, Aswan, Egypt; ^6^National Heart and Lung Institute, Imperial College, London, United Kingdom

**Keywords:** maximum entropy method, wave intensity analysis, Hypertrophic Obstructive Cardiomyopathy, coronary artery, wave identification

## Abstract

**Background:** Wave intensity analysis is useful for analyzing coronary hemodynamics. Much of its clinical application involves the identification of waves indicated by peaks in the wave intensity and relating their presence or absence to different cardiovascular events. However, the analysis of wave intensity peaks can be problematic because of the associated noise in the measurements. This study shows how wave intensity analysis can be enhanced by using a Maximum Entropy Method (MEM).

**Methods:** We introduce a MEM to differentiate between “peaks” and “background” in wave intensity waveforms. We apply the method to the wave intensity waveforms measured in the left anterior descending coronary artery from 10 Hypertrophic Obstructive Cardiomyopathy (HOCM) and 11 Controls with normal cardiac function. We propose a naming convention for the significant waves and compare them across the cohorts.

**Results:** Using a MEM enhances wave intensity analysis by identifying twice as many significant waves as previous studies. The results are robust when MEM is applied to the log transformed wave intensity data and when all of the measured data are used. Comparing waves across cohorts, we suggest that the absence of a forward expansion wave in HOCM can be taken as an indication of HOCM. Our results also indicate that the backward compression waves in HOCM are significantly larger than in Controls; unlike the forward compression waves where the wave energy in Controls is significantly higher than in HOCM. Comparing the smaller secondary waves revealed by MEM, we find some waves that are present in the majority of Controls and absent in almost all HOCM, and other waves that are present in some HOCM patients but entirely absent in Controls. This suggests some diagnostic utility in the clinical measurement of these waves, which can be a positive sign of HOCM or a subgroup with a particular pathology.

**Conclusion:** The MEM enhances wave intensity analysis by identifying many more significant waves. The method is novel and can be applied to wave intensity analysis in all arteries. As an example, we show how it can be useful in the clinical study of hemodynamics in the coronary arteries in HOCM.

## 1. Introduction

Wave Intensity Analysis (WIA) is useful for analyzing arterial hemodynamics, particularly in the coronary arteries. Many different types of waves can exist in the elastic arteries (1), but the dominant wave that we are considering is the elastic axial wave; which exchanges energy between the kinetic energy of the blood flow and the potential energy in the elastic arterial wall (2, 3). Wave intensity is defined as the product of the pressure and velocity differences across a wave front, *dI* = *dPdU*, and represents the energy flux per unit area carried by the wave (4, 5).

Waves propagate in both the forward and backward direction in the arteries; wave intensity is positive for forward waves and negative for backward waves. Wave intensity calculated from the measured pressure and velocity at a given site in an artery is the sum of the forward and backward wave intensities; so that the wave intensities of simultaneous forward and backward waves tend to cancel each other. If the wave speed is known, it is possible to separate the forward and backward wave intensities.

**Figure 1:**
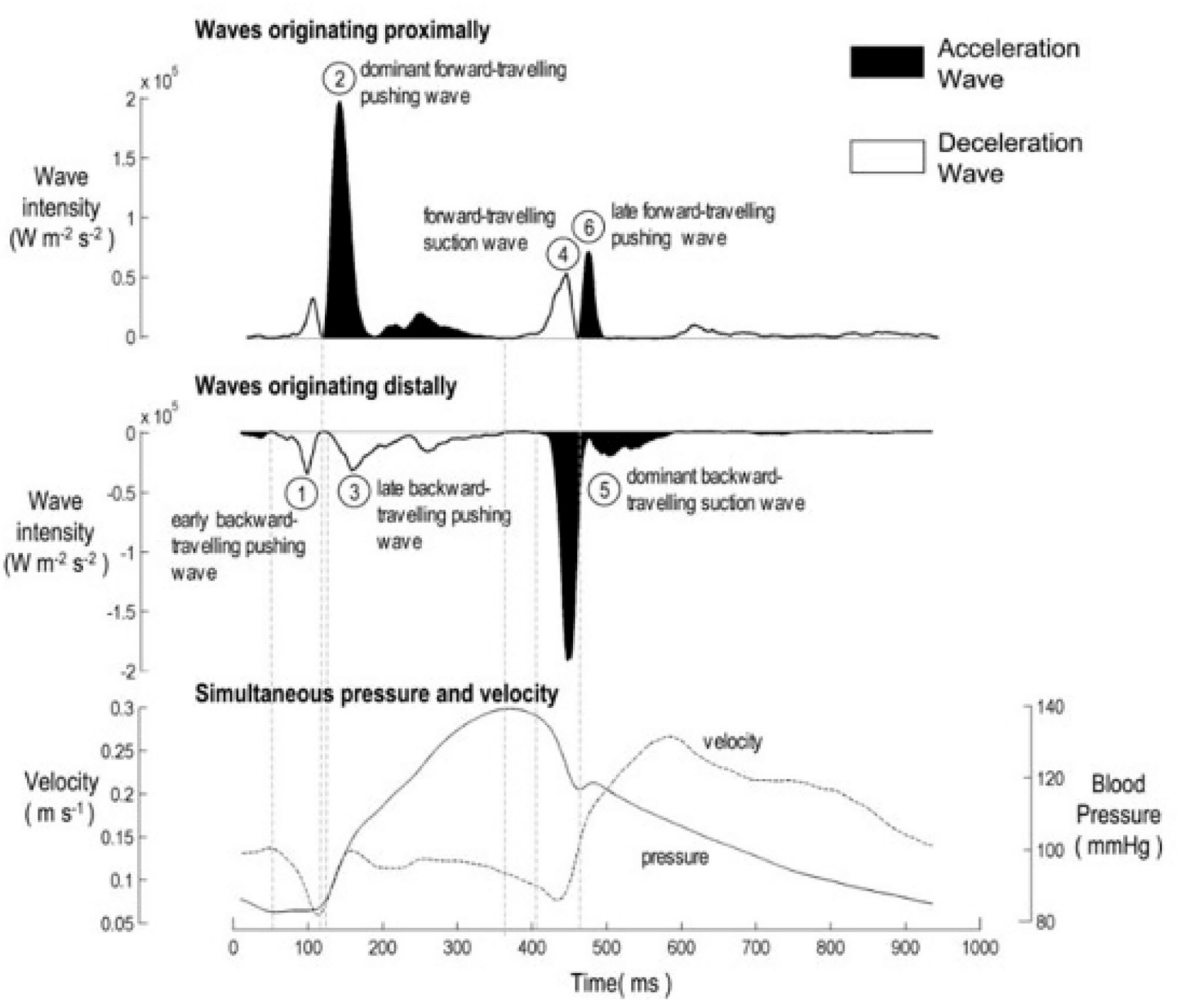
The six characteristic waves in healthy unobstructed coronary arteries, adapted from Davies et al. (6). **(Top)** Forward wave intensity originating near the aorta. **(Middle)** Backward wave intensity originating near the microcirculation. **(Bottom)** Total pressure and velocity waveforms used in the calculation of wave intensity analysis. The peaks are identified as 1: Early Backward Compression Wave (*BCW*_0_); 2: Forward Compression Wave (*FCW*_1_); 3: Backward Compression Wave (*BCW*_1_); 4: Forward Expansion Wave (*FEW*_2_); 5: Backward Expansion Wave (*BEW*_2_); 6: Late Forward Compression Wave (*FCW*_2_).

Separated wave intensity analysis measures both the magnitude and the direction of waves in the artery. Figure 1 is an example of the separated wave intensity calculated from pressure and flow measurements in a human circumflex artery (6). The ability of wave intensity to separate the effects of forward and backward waves is one of its main advantages, particularly in coronary arteries where the contraction of the myocardium directly causes both forward and backward waves.

Much of the clinical application of wave intensity analysis has involved identifying waves from the peaks in the wave intensity and relating these waves (or their absence) to events in the ventricle or alterations in the downstream sites of reflection (2, 7). In Figure 1, the authors have identified 6 peaks and identified them by their timing, their direction of travel and their affect on the blood velocity (acceleration or deceleration). Comparing wave intensity measurements from diseased and normal patients has been useful in determining the affect of different pathologies on arterial hemodynamics (7).

In other retrospective clinical studies, the relative magnitude of wave intensity peaks has been shown to be a significant predictor of cardiovascular risk (3). In all of these studies, peaks in the wave intensity have been identified “by eye,” which is straightforward for the larger peaks but becomes problematic for smaller peaks. As seen in Figure 1, there are many local maxima in all wave intensity measurements. In this paper, we explore the use of information theory, in particular the Shannon entropy of the wave intensity signal, to distinguish “peaks” from “background” thereby enhancing wave intensity analysis.

The analysis of wave intensity peaks can be problematic because of the associated physiological noise in the measurements (e.g., heart rate variations) as well as the inevitable noise in the measurement of pressure and velocity. We introduce a method for differentiating between “peaks” and “background” in the wave intensity waveform based on the Maximum Entropy Method (MEM). The idea is very simple in concept and we believe its application to wave intensity analysis is novel.

We conclude the paper by comparing wave intensity waveforms measured in the left anterior descending coronary artery in a small cohort of patients with Hypertrophic Obstructive Cardiomyopathy (HOCM) and a small cohort of patients with normal cardiac function. This study demonstrates how MEM can enhance the identification of waves from the wave intensity analysis of pressure and flow in coronary arteries.

## 2. Materials and Methods

### 2.1. Maximum Entropy Method

Information theory was introduced in the study of communications (8) but can be extended to any subject that involves probability and statistics. Central to information theory is the concept of *entropy* (usually called Shannon entropy to distinguish it from thermodynamic entropy). The probability of any event *p* is a measure of our expectation that it will occur in any trial; when *p* is small the event will occur infrequently, and when it is large it will occur frequently. Obversely, the reciprocal $\frac{1}{p}$ can be thought of as a measure of our uncertainty about the occurrence of an event. For subtle axiomatic reasons, Shannon defined uncertainty as $\log\frac{1}{p}$, and entropy as the expected outcome over all possible events *n* = 1…*N*.

(1) $$H=\sum_{n=1}^{N}p_{n}\log\left(\frac{1}{p_{n}}\right)=-\sum_{n=1}^{N}p_{n}\log p_{n}$$

Thus, the entropy *H* is the average uncertainty about the outcome of a trial. *p* is non-dimensional and so *H* is non-dimensional, however its magnitude depends upon the base of the logarithm used; when logarithm to the base 2 is used, as in this study, entropy is given in “bits.”

The link between entropy and information is the realization that when a trial is performed and the outcome is known uncertainty is removed, and so the information that we have received from the trial is equal to the entropy before the trial was performed. Despite its name, information in this sense is not a measure of the value of the outcome; the entropy of a Shakespearean sonnet is exactly equal to the entropy of the same letters jumbled up in any random order.

The Maximum Entropy Method (MEM) involves partitioning data in a way that maximizes the entropy of the data (9). Its utility has been demonstrated in many areas such as the treatment of missing data in clinical trials, artificial intelligence, computer vision, and the optimal categorization of data.

In this study, we investigate the use of MEM to separate a measured wave intensity waveform into “peaks” and “background” using the Bayesian statistical principle; the probability distribution function that best represents the categorized data is the one with the largest entropy. This reduces the problem to finding the optimal threshold between peaks and background. This separation is particularly important in wave intensity analysis because of the inherent noisiness of wave intensity, which is defined as the product of two noise-enhancing derivatives.

The main problem in applying MEM is that we do not have prior knowledge of the probability distribution of the data; the probability must be estimated from the observed data. In our study, the data *X* is a time series of wave intensities *x*_*n*_ measured at discrete time intervals *t*_*n*_. The probability of each *x*_*n*_ is estimated from the histogram of data *h*, which lies within bins *b*. *h*_*j*_ is the number of data points that fall within each bin *b*_*j*_, and the sum of *h*_*j*_ is equal to N. Thus, $p_{j}=\frac{h_{j}}{N}$ is an estimate of the probability, which enables us to calculate the entropy of the signal using:

(2) $$H=-\sum_{j=1}^{J}p_{j}\log p_{j}$$

where *J* is the number of bins in the histogram; determined in our case by rounding the square root of the length of data. When the data *X* are categorized into two categories by a threshold *x*_*T*_: peaks Π (*x*_*n*_ ≥ *x*_*T*_) and background Φ (*x*_*n*_ < *x*_*T*_), the total entropy *H*_*T*_ is calculated using entropy *H*_Π_ and *H*_Φ_:

(3) $$H_{T}=H_{\Pi }+H_{\Phi }=-\sum_{j\in \Pi }p_{j}\log p_{j}-\sum_{j\in \Phi }p_{j}\log p_{j}$$

Since the entropy is a measure of the information in the signal, MEM asserts that the optimal threshold is the one that maximizes the total entropy of the partitioned data. Finding the maximum can be done iteratively or, more simply, by calculating the maximum net entropy over an equi-spaced array of thresholds and determining the maximum by interpolation.

### 2.2. Application of Maximum Entropy Method to Wave Intensity Analysis

Wave intensity is an inherently noisy signal. It is the product of differences which tends to emphasize the inevitable measurement noise in the pressure and velocity, and it is sensitive to physiological “noise” such as breathing and heart rate variations. Also, the use of an estimated local wave speed in the separation of wave intensity into its forward and backward components introduces systematic error which is very difficult to model. Given the level of noise and the difficulty in analyzing wave intensity peaks, MEM provides a convenient way to separate significant peaks from the noisy background.

**Figure 2:**
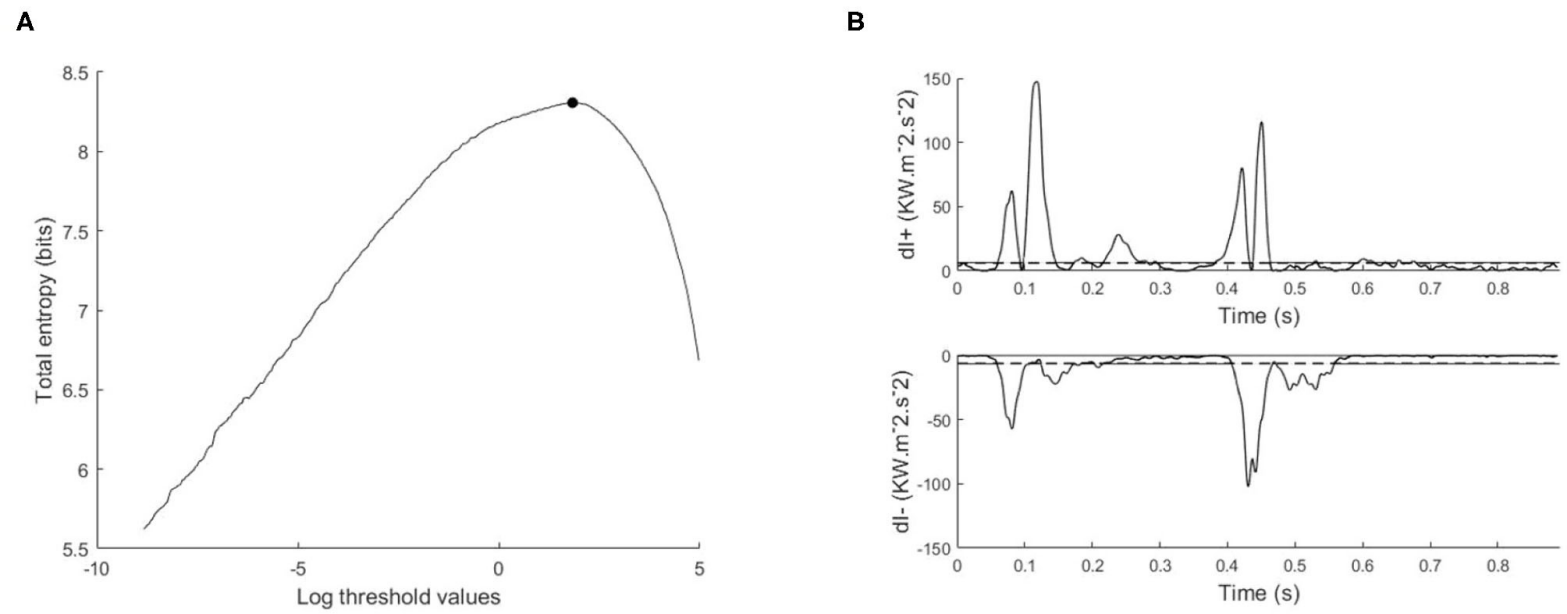
Illustration of maximum entropy method and its application to the wave intensity signal, **(A)** total entropy as a function of the thresholds calculated on the log transformed data, with the optimal threshold indicated with a black circle, **(B)** separated wave intensity waveforms; forward (*dI*_+_) and backward (*dI*_−_). The horizontal dashed line is the exponential of the optimal threshold.

Assuming that the noise is the same for the forward wave intensity (*dI*_+_) and the backward wave intensity (*dI*_−_), then the signal *X* = *dI*_+_ ∪ |*dI*_−_| is categorized using the same threshold (*dI*_*T*_), where “peaks” are ≥ *dI*_*T*_, and “background” is < *dI*_*T*_. The optimal threshold is defined as the threshold that maximizes the total entropy *H*_*T*_(*dI*_*T*_), see Figure 2.

**Figure 3:**
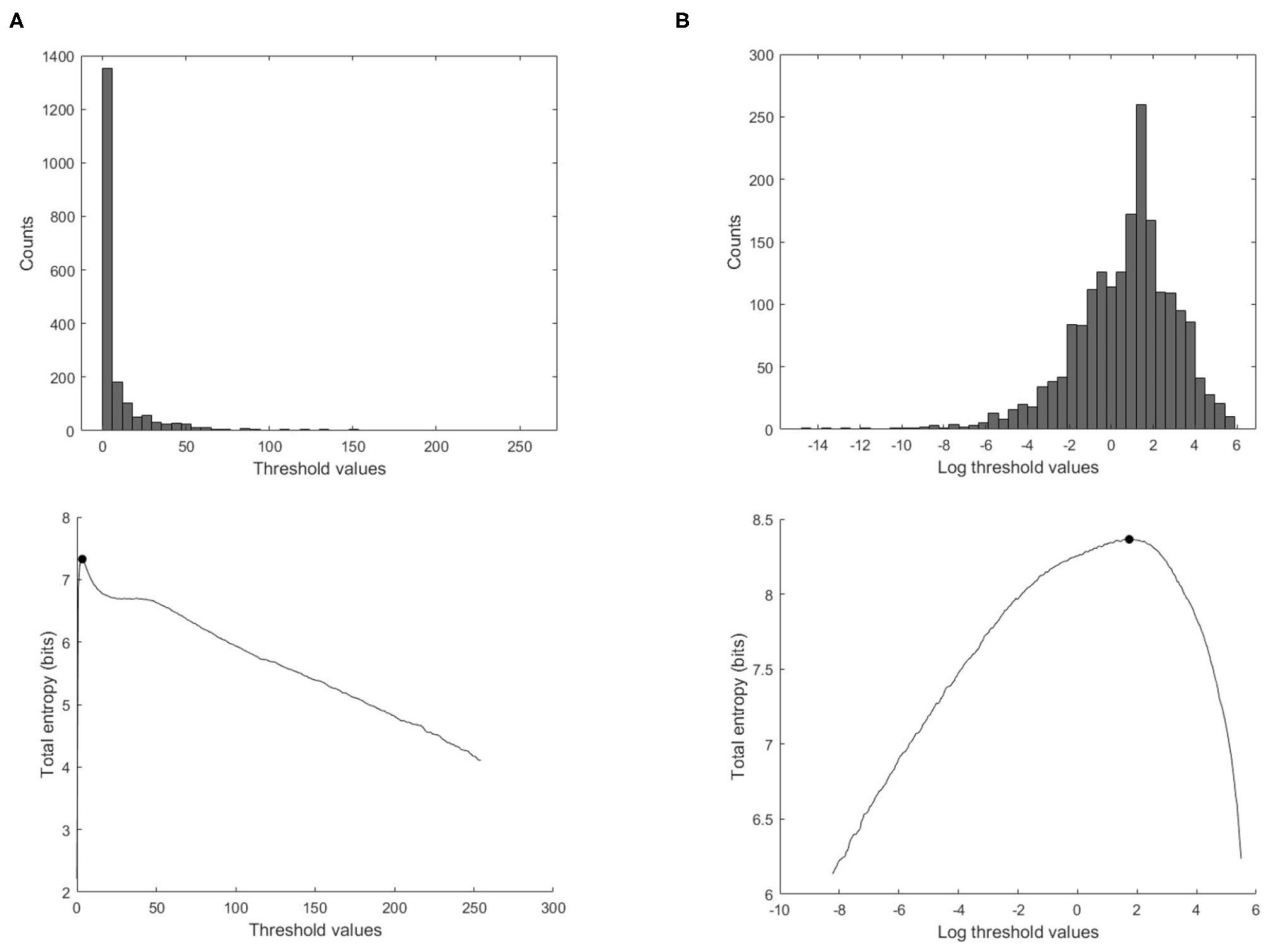
Effect of using the log transform data on the calculation of the total entropy. **(Top)** Histogram of the ensemble wave intensity data of a control subject. **(Bottom)** Total entropy as a function of the threshold calculated on the wave intensity data, with the optimal threshold indicated with the black solid circle, **(A)** threshold values in the original units, **(B)** threshold values transformed to the logarithmic values.

From our experience in applying MEM to wave intensity waveforms, there are two techniques that greatly increase the robustness and reproducibility of the method. The first technique arises from the highly skewed nature of the distribution of clinical wave intensity measurements, see Figure 3. The exponential shape of the histogram introduces a number of difficulties in estimating the probabilities. By introducing the log transform *X* = *log*(*dI*), the resulting histogram is much more normally distributed, and the results of the entropy calculations are much more robust. All of our results are obtained by performing the entropy calculations on the log transformed data and then transforming the optimal threshold back to the original units by taking its exponential.

**Figure 4:**
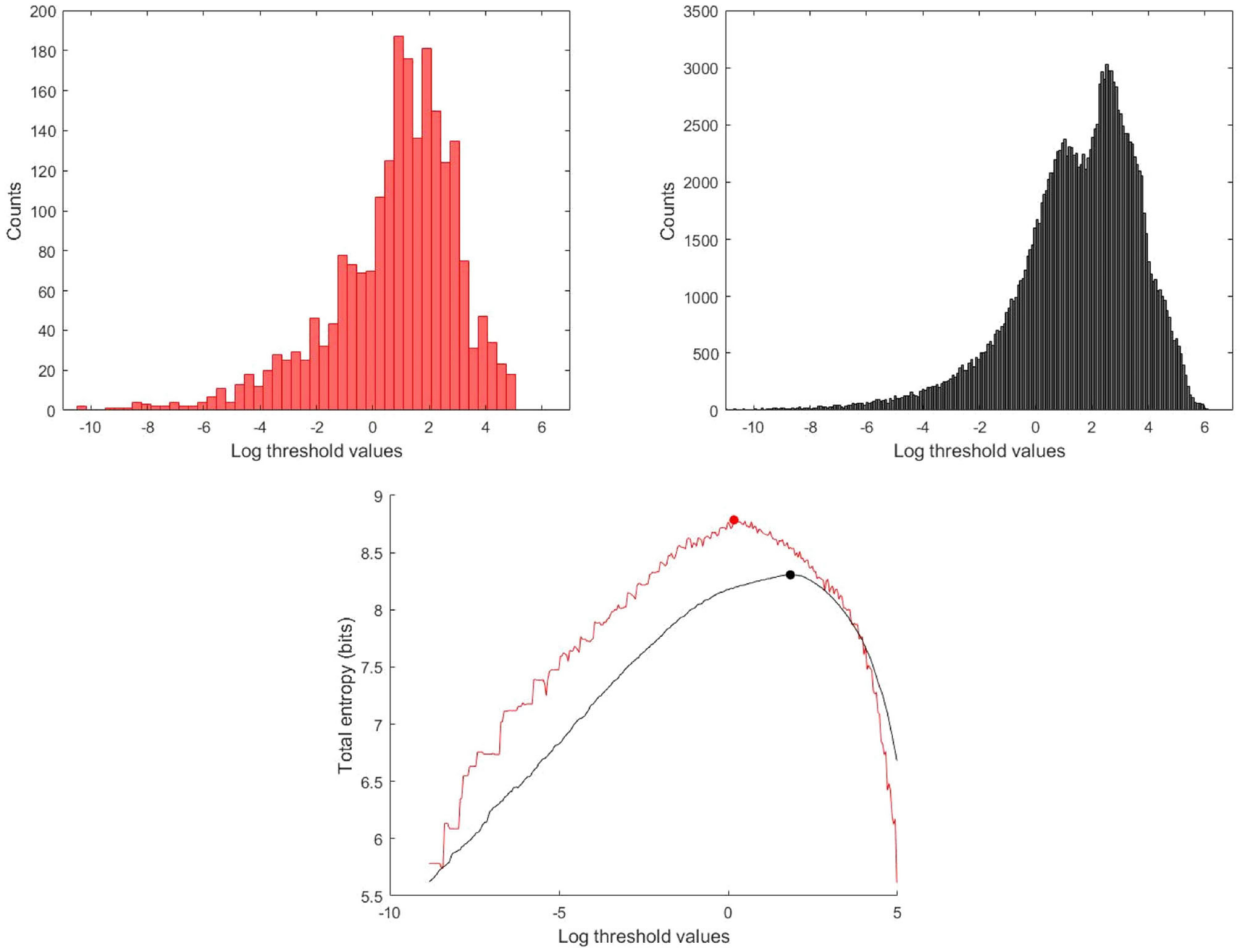
Histogram and total entropy as a function of the thresholds for the log transformed data, **(Red)** Histogram and total entropy calculated using the ensemble averaged wave intensity signal, **(Black)** calculated using the total wave intensity data.

Second, it is best to perform the analysis using all of the data rather than the ensemble averaged wave intensity. Estimating the underlying probability density function used in calculating the entropy from the data itself is a fundamental problem in statistics. We tried a number of methods, including kernel smoothing methods, but found that estimating the probability from the more highly populated histograms using the total log transformed data is superior for our data, see Figure 4.

### 2.3. Data Acquisition

WIA was performed using the simultaneously measured pressure and flow via a 0.014 inch diameter velocity and pressure sensor wire (Combowire, Volcano Corporation, San Diego, California) positioned appropriately in the proximal left anterior descending coronary artery in 10 patients (age 30 ± 8 years old) with symptomatic HOCM (10–13) prior to undergoing myectomy at Aswan Heart Center-Magdi Yacoub Heart Foundation; and in 11 normal Controls were patients who presented at St. Mary's Hospital for coronary angiography and were found to have structurally normal hearts with absence of valvular pathology or coronary artery obstruction (6). All patients and controls signed consent forms following ethical approval from the ethical committees at Aswan Heart Center-Magdi Yacoub Heart Foundation or St. Mary's Hospital, as appropriate.

The output from the Combowire console of the recordings of pressure, velocity, and ECG were digitized, processed offline and imported into MATLAB (The MathWorks, Inc., Natick, Massachusetts) for the post-processing using a custom-made MATLAB program (6, 7).

For details about the signal processing and the physics behind the analysis, see (5). Briefly, the measured pressure and velocity data *P* and *U* were converted into pressure and velocity differences *dP* and *dU* using a Savitsky-Goalay filter (2nd order, sliding frame of 11 samples), where ‘d’ is defined as ‘d/dt’, and dt is the sampling time, to accommodate different data sampling rates. The net wave intensity *dI* = *dPdU* can be useful but does not differentiate between forward and backward traveling waves. This separation requires knowledge of the local wave speed *c* which can be estimated by the sum of squares method (calculated over an integral number of cardiac cycles)

(4) $$c=\frac{1}{\rho }\sqrt{\frac{\sum \left(dP^{2}\right)}{\sum \left(dU^{2}\right)}}$$

where the density of blood ρ is assumed to be 1,050 kgm^−3^ (3, 6). From the water hammer equation

(5) $$dP_{\pm }=\pm \rho c(dU_{\pm })$$

and the assumption that the forward (+) and backward (–) waves are additive, *dP* = *dP*_+_+*dP*_−_ and *dU* = *dU*_+_+*dU*_−_, the forward and backward pressure and velocity changes are (2, 5)

(6) $$dP_{\pm }=\frac{1}{2}(dP\pm \rho cdU)$$

(7) $$dU_{\pm }=\pm \;\frac{1}{2\rho c}(dP_{\pm }\;\rho cdU)$$

Finally, the forward and backward wave intensities are calculated as

(8) $$dI_{\pm }=dP_{\pm }dU_{\pm }.$$

### 2.4. Identification of Significant Wave Intensity Peaks

The separated wave intensities can be thought of as the magnitude of the forward and backward wave fronts that are passing the measurement site at any particular time. Peaks in the separated wave intensities are usually referred to, rather confusingly, as “waves.” The direction of these waves is determined by the sign of the wave intensity; positive for forward waves and negative for backward waves. The nature of the waves is determined by the sign of *dP*; waves with positive *dP* are compressive waves and those with negative *dP* are expansion waves. There are therefor four types of waves in the arteries: Forward Compression Waves (FCW), Forward Expansion Waves (FEW), Backward Compression Waves (BCW) and Backward Expansion Waves (BEW). The effect of the waves on the velocity are determined by the sign of *dU*; waves with positive *dU* are acceleration waves and those with negative *dU* are deceleration waves. Both FCW and BEW are acceleration waves and both FEW and BCW are deceleration waves.

The timing of the waves is indicated by subscripts 0, 1 and 2 depending upon the phase of the cardiac cycle when they appear. The three phases are marked by the R-wave of the ECG (the iso-volumic contraction phase), the start of the rapid rise in pressure (early systole) and the start of the rapid rise in velocity (late systole/early diastole). We note that these phases are unique to the coronary arteries; in other arteries the start of the rapid rise in velocity is simultaneous with the start of the rapid rise in pressure. Occasionally other waves of the same nature occur in the same phase and the subsequent waves are denoted by appending the subscripts “a” and “b.”

The ability to relate the magnitude, the direction and the timing of the waves to events, both upstream and downstream, in the coronary arteries is the main value of wave intensity analysis. For each significant wave, as determined by the MEM analysis, we measure the peak value, time of the peak, wave duration, wave energy (time integral of power) and the percentage of the wave energy (wave energy fraction) calculated from dividing each wave energy by the cumulative wave energy of all the waves in that subject. In this study, we report results for measurements in 21 subjects (11 Controls, 10 HOCM).

### 2.5. Statistical Analysis

Data are expressed as mean ± standard deviation for different parameters. The wave intensity data were calculated from a single, simultaneous measurement of pressure, velocity and ECG for each subject. For data comparison between HOCM and Controls, we used the Mann-Whitney U test. A p ≤ 0.05 is considered statistically significant, which indicates that the observations from HOCM patients differ from those from Controls.

## 3. Results

The primary purpose of this paper is to introduce the application of MEM for the identification of significant waves in an artery by the detection of significant peaks in the wave intensity waveform. This is relevant to all wave intensity analysis. The secondary purpose is to present some preliminary results from a small study of HOCM patients. These patients are scheduled for myectomy and will eventually be part of a larger study measuring the clinical and mechanical effects of myectomy. The methodological results will use representative data from HOCM and Control subjects. The more clinical results will compare the MEM results in a cohort of 21 subject, 10 HOCM and 11 Controls.

### 3.1. Methodological Results

#### 3.1.1. Estimation of the Probability Density Function of Wave Intensity

Calculation of the entropy of a digitized signal depends on the probability of each measurement. In clinical measurements, this probability is not known and must be inferred from the data itself. Due to the highly skewed distribution of wave intensity data (dI) observed in most cases, we have explored transforming the distribution of the data from a skewed distributed data to a more Gaussian distribution using the log transform of the data “log (dI)” (see Figure 3).

#### 3.1.2. Ensemble Averaged vs. Total Wave Intensity Data

Because of the quasi-periodic nature of the cardiac cycle, most dynamic measurements of cardiovascular parameters are presented as a single “representative” beat or, preferably, as an ensemble average of the measurements over an ensemble of beats. Most wave intensity results, for example those shown in Figure 1, are calculated from the ensemble averaged *P* and *U*. In our study, we determined that the ensemble average of the instantaneous wave intensity is very close to the wave intensity calculated from the ensemble average *P* and *U*. Therefore, all of our preliminary studies of MEM were done using the ensemble average *dI*. The results were encouraging but not very robust, being very sensitive to various parameters in the calculations and producing an unacceptable number of outliers. We then tried MEM using the instantaneous *dI* calculated from all of the measured *P* and *U*, and found the results to be much more satisfactory. Figure 4 shows the histograms and the maximum total entropy for the categorized data using the two methods: wave intensity calculated from the ensemble averaged *P* and *U* or from the total *P* and *U* data.

### 3.2. Clinical Results

Wave intensity analysis provides a quantitative description of the timing, magnitude and direction of the waves that determine the pressure and velocity in an artery. This is particularly useful in the coronary arteries because the contraction of the ventricle causes disturbances to the distal intra-myocardial blood vessels which profoundly affect coronary artery hemodynamics. Previous studies have concentrated on the main waves, manifested as large peaks in the wave intensity waveforms, comparing the differences between normal and pathological conditions such as HOCM (7). We seek to extend this work by identifying smaller but still significant waves using a MEM to differentiate between peaks and background in the clinically measured wave intensity waveforms.

**Figure 5:**
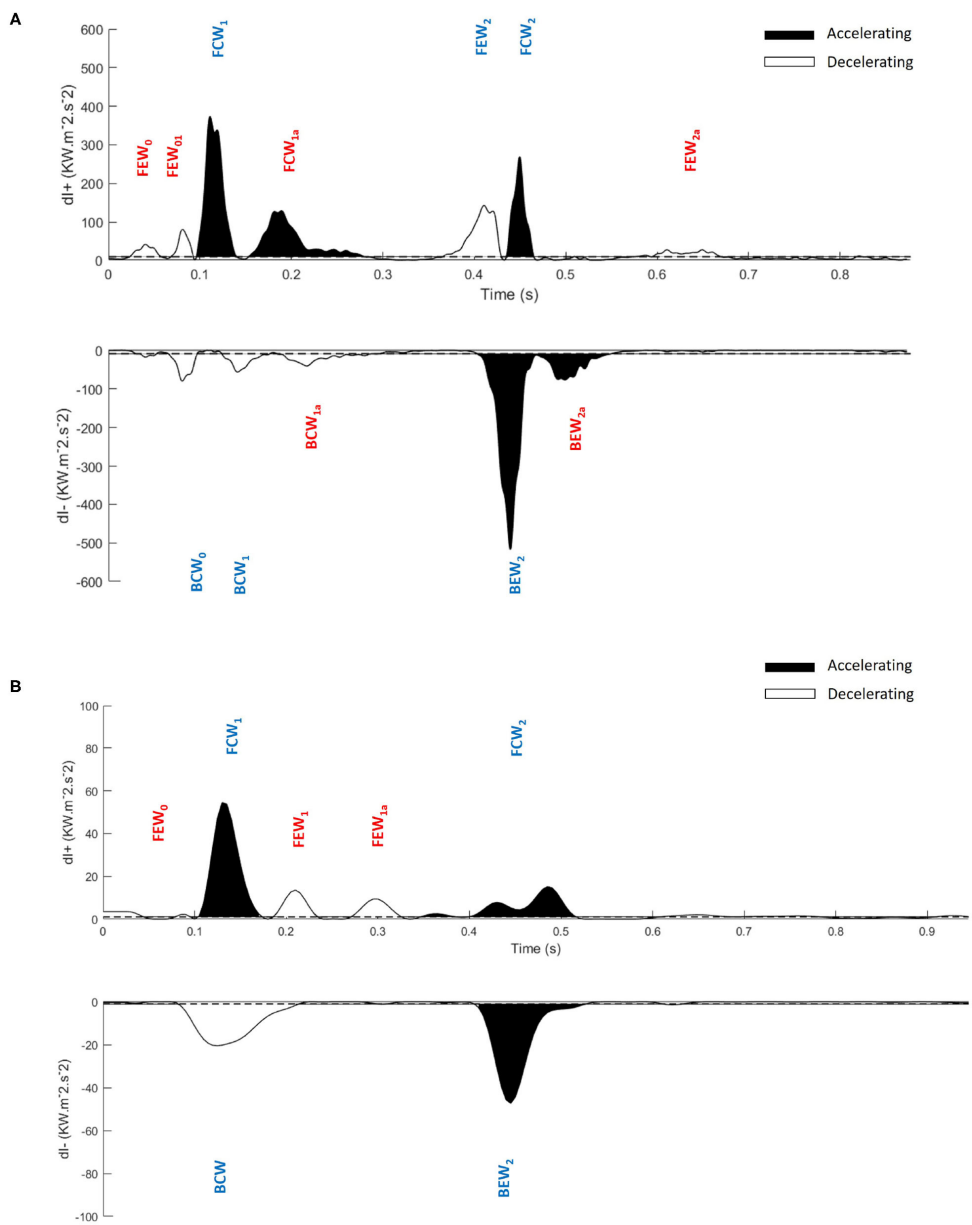
Application of the maximum entropy method to wave intensity waveforms and identification of significant peaks. Significant peaks in the separated ensemble average wave intensity waveforms; forward (*dI*_+_) and backward (*dI*_−_), **(A)** Control subject, **(B)** Hypertrophic Obstructive Cardiomyopathy patient. FEW, Forward Expansion Wave; FCW, Forward Compression Wave; BCW, Backward Compression Wave; BEW, Backward Expansion Wave. The blue colored waves are the dominant waves, and red colored waves are the secondary waves. Note the difference in scales between **(A,B)**.

The benefits of MEM can be seen by comparing Figure 1, a previously published wave intensity analysis in a normal subject, with Figure 5, a normal subject in our study. The previous study identified 6 waves while using MEM we have identified 6 additional significant waves, most of which are present in Figure 1 but presumably considered to be noise.

In this study, we introduce a systematic nomenclature for waves in coronary arteries which may be useful in future studies. Our cohort of subjects is too small to reach any clinical results but we do present preliminary results for guidance and the statistical information necessary to properly power future clinical studies. Our approach is to divide the waves into dominant and secondary waves. We further divide the secondary waves into categories depending on the frequency that we observe them in our subjects, discussing their probable origin and their possible value in diagnosis.

The waves are named using the following convention:

**F/B** for Forward/Backward where forward is defined by the mean direction of blood flow.
**C/E** for Compression/Expansion depending on whether the pressure Increases/Decreases across the wave.
_**0/1/2**_ depending on the phase of the cardiac cycle in which they appear.
_***a*/*b*/…**_ refers to the successive individual waves with the same nature, that are occasionally observed.

For instance, *FCW*_1*a*_ is a Forward Compression Wave that follows *FCW*_1_, which appears in the period between the rapid rise of pressure in early systole and the rapid rise of velocity in late systole.

#### 3.2.1. Dominant Waves

As expected, the total wave energy was much larger in Controls than in HOCM patients; the average over Controls was 27 ± 9 kWm^−2^s^−1^ compared to 15 ± 8 kWm^−2^s^−1^ in HOCM. Statistically, this difference is significant with *p* < 0.01. To enable comparisons of individual waves between the two groups, we define the relative wave energy as the wave energy in a single wave, the time integral of the wave energy over the duration of the peak, divided by the total wave energy in that subject.

Waves are classified as “dominant” if they are present in all the subjects and their relative wave energy is >3%. The properties of the individual dominant waves are given in Table 1. The classification is essentially independent of the MEM analysis and comparison of Figure 1 and Table 1 shows that the dominant waves correspond to the numbered waves identified in previous studies (2, 6, 7, 14).

**Table 1:** The dominant waves.

**Dominant waves:**								
**Wave**	**# with wave**		**Wave energy**			**Wave energy fraction**		
	**Control**	**HOCM**	**Control**	**HOCM**	***P*** **-value**	**Control**	**HOCM**	***P*** **-value**
	**(** ***n*** **= 11)**	**(** ***n*** **= 10)**	**(** ***n*** **= 11)**	**(** ***n*** **= 10)**		**(** ***n*** **= 11)**	**(** ***n*** **= 10)**	
FCW_1_	11	10	6.7 ± 2.4	2.2 ± 1.3	<0.001	27 ± 10	15 ± 6	<0.001
FEW_2_	11	-	2.3 ± 1.3	-	-	8 ± 3	-	-
FCW_2_	11	10	3.4 ± 1.5	0.9 ± 0.5	<0.001	14 ± 5	6 ± 3	<0.001
BCW_0_	11	10	1.0 ± 1.0	5.5 ± 4.0	<0.001	4 ± 3	32 ± 10	<0.001
BCW_1_	11		1.4 ± 0.6			6 ± 3		
BEW_2_	11	10	8.1 ± 4.4	5.5 ± 2.9	0.1	29 ± 10	35 ± 9	0.1

*Wave energy (kWm^−2^s^−1^) and the wave energy fraction (%) for each wave are given as (mean ± standard deviation). HOCM: Hypertrophic Obstructive Cardiomyopathy*.

#### 3.2.2. Secondary Waves

The secondary waves are described in Table 2 where they are classified into four categories depending on their frequency of observation in our subjects:

**A** waves that are observed in more than 40% of both Controls and HOCM subjects.
**B** waves that are observed in more than 40% of Controls but not in HOCM.
**C** waves that are observed in more than 40% of HOCM patients but not in Controls.
**D** waves that are observed in <40% of both groups.

**Table 2:** The secondary waves.

**Secondary waves:**							
	**Wave**	**# with wave**		**Wave energy**		**Wave energy fraction**	
		**Control**	**HOCM**	**Control**	**HOCM**	**Control**	**HOCM**
		**(** ***n*** **= 11)**	**(** ***n*** **= 10)**	**(** ***n*** **= 11)**	**(** ***n*** **= 10)**	**(** ***n*** **= 11)**	**(** ***n*** **= 10)**
	FEW_0_	11	9	0.8 ± 1.1	0.6 ± 0.7	3 ± 4	3 ± 3
A	FEW_2*a*_	11	9	0.4 ± 0.5	0.2 ± 0.2	2 ± 2	2 ± 2
	FEW_2*b*_	7	5	0.2 ± 0.3	0.1 ± 0	1 ± 1	0 ± 0
	FCW_1*a*_	8	2	1.9 ± 1.9	0.1 ± 0.1	6 ± 5	1 ± 0
	BEW_2*a*_	5	1	0.8 ± 0.6	0.3	4 ± 3	3
B	FEW_01_	6	-	0.7 ± 0.7	-	2 ± 2	-
	BCW_1*a*_	6	2	0.6 ± 0.8	0.1 ± 0	2 ± 2	1 ± 0
	FCW_1*b*_	8	-	0.4 ± 0.3	-	3 ± 2	-
	FEW_1_	-	9	-	0.3 ± 0.2	-	3 ± 3
C	BEW_1_	-	4	-	0.2 ± 0.2	-	2 ± 2
	FCW_2*a*_	1	1	0.5	0.2	8	2
D	FEW_1*a*_	-	1	-	0.3	-	7
	BCW_2_	4	3	0.1 ± 0.1	0.2 ± 0.3	0 ± 0	3 ± 4

*The waves are divided into 4 subcategories based on their frequency of observation and their wave energy, as discussed in the text. Wave energy (kWm^−2^s^−1^) and the wave energy fraction (%) for each wave are given as (mean ± standard deviation). HOCM, Hypertrophic Obstructive Cardiomyopathy*.

The secondary waves observed in both Controls and HOCM are listed in Table 2 together with their frequency of observation, their average wave energy and wave energy fraction.

## 4. Discussion

### 4.1. Methodological Discussion

Our original efforts to apply MEM to wave intensity measurements were disappointing, probably because of the highly skewed distribution of *dI*, see Figure 3. Observing that the log transformed data, [log(*dI*)], exhibited a much more normal distribution, we applied the analysis to the log transformed data which gave reasonable and robust results. Thus, the probability density is estimated from the log transform of the observed data using a histogram function for regularly spaced thresholds over the range [*T*_*min*_, *max*(*dI*)]. *T*_*min*_ is determined from the histogram of all of the data as the smallest bin containing more than 1 count. Tests indicated that the total entropy results are not sensitive to slight changes in the value of *T*_*min*_.

Similarly, we found that MEM applied to wave intensity calculated for an ensemble averaged beat gave less reliable results than MEM applied to the wave intensity calculated from all of the instantaneous measurements of *P* and *U*. This is shown for a typical subject in Figure 4. The total entropy *H*_*T*_ calculated for the total wave intensity data (black) is much smoother than *H*_*T*_ calculated for the ensemble averaged data (red). More importantly, the maximum which is used to find the optimal threshold is shifted to a smaller value when the ensemble averaged data are used. The explanation for this shift is not obvious to us but it was observed consistently in our subjects. We therefore recommend that MEM should be used with the total rather than the ensemble averaged wave intensity data.

### 4.2. Clinical Discussion

We have used MEM to categorize the data and separate the significant peaks from the non-peaks. We refer to the noisy non-informative peaks as “background” because they include physiological noise, instrumental noise and any other unknown noise. We have applied MEM to WIA in one clinical application, HOCM, where wave intensity is thought to have predictive value of risk (3) but it is frequently difficult to identify the significant peaks from the background. Because of the small size of our cohort (21 subjects), all of the clinical observations should be considered to be preliminary; providing pointers to future clinical studies.

For convenience, we have divided the measured waves into dominant and secondary waves. The dominant wave coincide almost exactly with the waves measured in previous studies (2, 6, 7, 14) and their data are in Table 1.

Our results confirm the observation that the wave *FEW*_2_ (i.e., the Forward Expansion Wave occurring in late systole/early diastole) that is observed in all Controls is not observed in any of the HOCM patients. This suggests that the absence of this wave could be taken as a clinical indication of HOCM. In this case, the MEM analysis could be useful because it gives a quantitative measure for the “absence” of the wave. Because of the small number of patients in our cohort, these results are preliminary and not clinically definitive. They do, however, suggest future clinical studies exploring the link between the absence of *FEW*_2_ and changes in the myocardium in HOCM.

Our results also indicate that the two backward compression waves, *BCW*_0_, occurring during the iso-volumic contraction period, and *BCW*_1_, occurring during early systole, which are distinct in Controls present as a summation wave in all of our HOCM patients. The wave energy of this summation wave in HOCM is significantly larger than the combined energy of the two separate waves in Controls. This is not the case for other dominant waves *FCW*_1_ or *FCW*_2_ where the wave energy in Controls is significantly higher than the wave energy in HOCM. This large BCW, a deceleration wave, is responsible for the majority of differences between the pressure and velocity waveforms in HOCM and Controls. Also, the reduced FCW in HOCM suggest that HOCM pathology could significantly derange the forward waves originating near the aorta, which suggests that the aorta may play a role in perturbing the coronary flow dynamics.

The relatively small secondary waves that are revealed using MEM are listed in Table 2 together with their properties, wave energy and fractional wave energy. For convenience we have divided these secondary waves into four categories depending on their frequency of observation in our subjects.

Category A waves are relatively small waves that are observed in both Controls and HOCM. Their wave energy in HOCM is approximately half that in Controls, which is comparable to the difference in total wave energy between HOCM and Controls. This suggests that their etiology is similar in both groups and their study may increase our understanding of the detailed interaction between the heart and the coronary arteries.

Category B waves are present in the majority of Controls and absent in almost all HOCM. This suggests that a study involving a larger number of subjects could find some diagnostic utility in the clinical measurement of these waves. Any future studies should be guided by the function of these waves. For example, the largest wave in Category B is *FCW*_1*a*_, which is a second forward compression wave that accelerates the blood during mid-systole and is generally missing in HOCM. Similarly, the second largest wave *BEW*_2*a*_ is a second backward expansion wave that accelerates blood during early diastole and is also present in most Controls but absent in most HOCM.

Category C waves are present in some of our HOCM patients but entirely absent in Controls. The largest of these waves is *FEW*_1_ which decelerates blood in early systole in some HOCM patients but is not present in any of our control subjects. Again, further study of this wave could provide supplementary evidence to the standard clinical tests for HOCM that may be useful in difficult cases or in differential diagnosis.

Category D waves are included only for completeness. They are not discussed because the observations are purely anecdotal.

## 5. Conclusion

The application of MEM to WIA for the identification of the significant peaks is novel and may enhance WIA in future study of coronary arteries. We demonstrate robust and reasonable results when we applied MEM to the log transformed wave intensity data, and smoother and more reliable results with the total rather than the ensemble averaged wave intensity data. Comparing the significant peaks in the wave intensity waveforms of Controls and HOCM patients, we have found an increase of backward compression waves and perturbed forward waves which altogether could perturb the coronary and aortic flow dynamics. We have also shown that some of the secondary waves identified using MEM are lost in HOCM patients, presumably due to the disease pathology, and that the appearance of *FEW*_1_ and *BEW*_1_ in HOCM patients could be predictor markers of HOCM. Thus, our provided data could help in elucidating the mechanisms involved in the complex interaction between coronary flow regulation and pathobiology of HOCM. However, the small number of subjects in our cohort is a serious limitation of our study and all our clinical results should be taken as preliminary.

## Data Availability Statement

The raw data supporting the conclusions of this article will be made available by the authors, without undue reservation.

## Ethics Statement

The studies involving human participants were reviewed and approved by St. Mary's Hospital Research Ethics Committee, Imperial College Healthcare National Health Service Trust, St. Mary's Hospital and Aswan Heart Centre Research Ethics Committee, Aswan Heart Centre, Magdi Yacoub Heart Foundation. The patients/participants provided their written informed consent to participate in this study.

## Author Contributions

NF, MY, and KP were involved in the conception of the research and the writing of the manuscript. NF and KP were involved in the computing, data analysis and other technical matters. NF, PS, and MY were involved in the data collection and other clinical matters. All authors approved the final version of the manuscript.

## Conflict of Interest

The authors declare that the research was conducted in the absence of any commercial or financial relationships that could be construed as a potential conflict of interest.

## Publisher's Note

All claims expressed in this article are solely those of the authors and do not necessarily represent those of their affiliated organizations, or those of the publisher, the editors and the reviewers. Any product that may be evaluated in this article, or claim that may be made by its manufacturer, is not guaranteed or endorsed by the publisher.
